# Supplementary material for: PFKFB3-Mediated Glycolysis Boosts Fibroblast Activation and Subsequent Kidney Fibrosis
Source: Cells. 2023 Aug 17;12(16):2081. doi: 10.3390/cells12162081 (PMC10453197; doi:10.3390/cells12162081)
Supplement: Supplementary file 1 [file cells-12-02081-s001.zip › cells-2557157-supplementary.pdf]

Supporting Figures

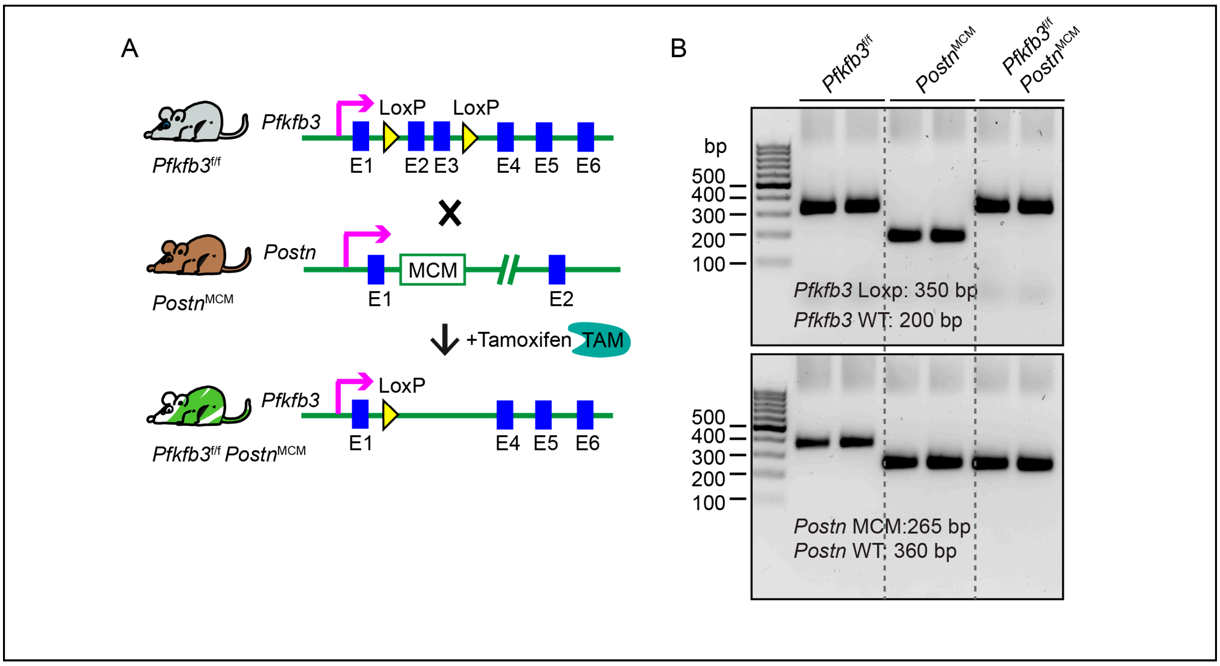

Supplementary Figure S1. Generation of myofibroblast-specific *Pfkfb3* deficient mice. **(A)** Schematic illustration of strategy to generate myofibroblast-specific *Pfkfb3* deficient mice. **(B)** Representative genotyping results for *Pfkfb3*$^{f/f}$ *Postn*^{MCM} and *Postn*^{MCM} mice.

Supporting Tables

Supplementary Table S1. Primers used for genotyping of murine strains.

Genotyping	Forward (5´-3´)	Reverse (5´-3´)
Pfkfb3 flox	GGAGTGGCCATTAGGTGGGGTT	CCAGCTTGGGCTACGCATTTAGTT
Postn MCM	TCTGTAAGGCCATCGCAAGCT	AATAAGTAAAACAGCTCCCCT
	GGTGGGACATTTGAGTTGCT	

Supplementary Table S2. Primers used for Quantitative RT-PCR.

Gene (Mouse)	Forward (5´-3´)	Reverse (5´-3´)
18S ribosomal	CTTAGAGGGACAAGTGGCG	ACGCTGAGCCAGTCAGTGTA
Pfkfb3	GATCTGGGTGCCCGTCGATCACCG	CAGTTGAGGTAGCGAGTCAGCTTC
Acta2	ATGCTCCCAGGGCTGTTTTCCCAT	GTGGTGCCAGATCTTTTCCATGT CG
Collagen 1	CCTCAGGGTATTGCTGGACAAC	CAGAAGGACCTTGTTTGCCAGG
Collagen 3	GACCAAAAGGTGATGCTGGACAG	CAAGACCTCGTGCTCCAGTTAG
Mmp2	CGGAAAGTGGAATCCTTGCAGG	AGCAGTGAGGTCAGGCTTGGAA
Mmp9	TTGAAGTCTCAGAAGGTGGAT	GCAGGAGGTCGTAGGTCAC
Fn	CCCTATCTCTGATACCGTTGTCC	TGCCGCAACTACTGTGATTCGG
Gene (Rat)	Forward (5´-3´)	Reverse (5´-3´)
Pfkfb3	CCAGCCTCTTGACCCTGATAAATG	TCCACACGCGGAGGTCCTTCAGAT
Acta2	GGAGATGGCGTGACTCACAA	AAGCACTGATGACGACTCGC
Fn	ACACGGTTTCCCATTACGCCAT	AATGACCACTGCCAAAGCCCAA

Supplementary Table S3. Antibodies used for Western blot and staining analysis.

Target protein	Company	Catalog #	Concentration
PFKFB3	Abcam	ab181861	1:1000
ACTA2	Santa Cruz Biotechnology	sc-32251	1:1000
Fibronectin (FN)	Abcam	ab2413	1:1000
Collagen I (COL1)	Novus	NB600-408	1:1000
Vimentin	Cell Signaling Technology	5741	1:1000
PCNA	Santa Cruz Biotechnology	sc-56	1:1000

p-SMAD2	Cell Signaling Technology	3208S	1:1000
SMAD2	Cell Signaling Technology	3122S	1:1000
α-tubulin	Cell Signaling Technology	3873S	1:1000
β-actin	Santa Cruz Biotechnology	sc-47778	1:1000
Cyclophilin B	Cell Signaling Technology	43603	1:1000
Anti-mouse IgG,<br>HRP-linked Antibody	Cell Signaling Technology	7076S	1:2000
Anti-rabbit IgG, HRP-linked antibody	Cell Signaling Technology	7074S	1:2000
ACTA2	Santa Cruz Biotechnology	sc-32251	1:100
Collagen IV (COL IV)	Abcam	ab6586	1:100
Collagen I (COL1)	Novus	NB600-408	1:100
Alexa Fluor 488-conjugated goat<br>anti-mouse secondary antibody	Invitrogen	A11001	1:250
Alexa Fluor 594-conjugated goat<br>anti-rabbit secondary antibody	Invitrogen	A11012	1:250
